# *Streptococcus agalactiae* and *Prototheca* spp. induce different mammary gland leukocyte responses in Holstein cows

**DOI:** 10.3168/jdsc.2022-0216

**Published:** 2022-05-21

**Authors:** S. Pegolo, A. Toscano, V. Bisutti, D. Giannuzzi, A. Vanzin, A. Lisuzzo, F. Bonsembiante, M.E. Gelain, A. Cecchinato

**Affiliations:** 1 Department of Agronomy, Food, Natural Resources, Animals and Environment (DAFNAE), University of Padova, Viale dell' Università 16, 35020, Legnaro PD, Italy; 2 Department of Animal Medicine, Productions and Health, University of Padua, Viale dell' Università, 16, 35020, Legnaro PD, Italy; 3 Department of Comparative Biomedicine and Food Science (BCA), University of Padua, Viale dell' Università 16, 35020, Legnaro PD, Italy

## Abstract

In this study, we investigated the association between natural subclinical intramammary infection (IMI) caused by *Streptococcus agalactiae* and *Prototheca* spp. and milk differential cell counts assessed by cytofluorimetric analysis in Holstein cows. After an initial bacteriological screening on 188 animals and a second assessment carried out 2 wk later aimed at confirming the bacteriological status, we collected milk samples from 47 animals and performed (1) milk composition analyses; (2) somatic cell counts and differential somatic cell counts (DSCC); and (3) cytofluorimetric analyses. Before statistical analyses, animals with co-infections were filtered out. Bacteriological status (negative, positive for *Strep. agalactiae*, or positive for *Prototheca* spp.) significantly affected the investigated traits. Compared with culture-negative samples, those that were positive for *Strep. agalactiae* and *Prototheca* spp. had higher SCS (+61% and +49%, respectively), DSCC (+4% and +19%, respectively), log polymorphonuclear neutrophil (PMN)-lymphocyte (LYM) counts (+59% and +71%, respectively), and log macrophage (MAC) counts (+63% and +72%, respectively). The individual leukocyte populations determined by cytofluorimetric analysis confirmed that mastitis infection increased the proportion of PMN in the milk samples compared with culture-negative samples, particularly when caused by *Strep. agalactiae* (+51%). In the case of MAC, the 2 pathogens behaved in opposite ways: *Strep. agalactiae* increased MAC by 41%, whereas *Prototheca* decreased MAC by 25%. *Prototheca* infection strongly increased the proportion of total T lymphocytes (TL; +87%) and T-helper lymphocytes (+83%). Accordingly, the (PMN+MAC):TL ratio increased with *Strep. agalactiae* infection (+95%) and decreased with *Prototheca* infection (−43%) compared with culture-negative samples. These results suggest the prevalence of an adaptive immune response and chronic inflammation in *Prototheca* infection, and an innate immune response to *Strep. agalactiae.* This knowledge might provide an important contribution to the development of novel and effective diagnostics and therapeutics.

## Highlights

• *Streptococcus agalactiae* increased polymorphonuclear neutrophils in milk samples.
• *Prototheca* infection greatly increased total T lymphocytes and T-helper lymphocytes in milk samples.
• *Prototheca* spp. trigger an adaptive immune response and chronic inflammation.

## Graphical Abstract

**Figure:**
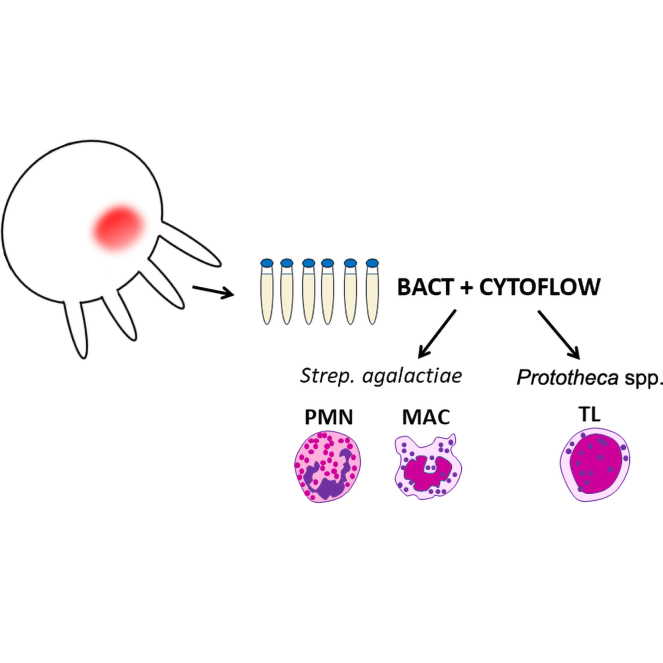
**Summary:** Further insights into the role of mammary gland leukocyte populations can help our understanding of the mechanisms underlying the response to mastitis infection in dairy cattle. We investigated the association between natural subclinical intramammary infection from *Streptococcus agalactiae* and *Prototheca* spp. and milk differential cell counts assessed by cytofluorimetric analysis in Holstein cows. *Prototheca* spp. infection induced an adaptive immune response and chronic inflammation mediated by T lymphocytes (TL) in the bovine mammary gland, whereas *Streptococcus agalactiae* infection induced an innate immune response mediated by neutrophils (PMN) and macrophages (MAC). This information is useful for the development of novel and effective diagnostics and therapeutics at the farm level.

Somatic cell count is a widely used method for detecting an inflammatory reaction in the mammary gland, whereas bacteriological analysis is required to detect and identify a pathogen. Recent studies have shown that assessment of inflammatory and bacteriological statuses is not always reliable because of the possibility of false-negative bacteriological results and infections without a concomitant increase in SCC (Schwarz et al., 2010; Bobbo et al., 2017; Pegolo et al., 2021, 2022).

Complementing the well-established SCC, cell differentiation provides a more accurate assessment of udder health status (Pilla et al., 2013). Differential SCC (**DSCC**) represents the combined proportions of lymphocyte (**LYM**) and neutrophil (**PMN**) populations, which have different roles in the mammary gland immune response (Lindmark-Månsson et al., 2006; Oviedo-Boyso et al., 2007; Schwarz et al., 2011). Damm et al. (2017) proposed using an automated milk analyzer and DSCC for routine mastitis screening, and Wall et al. (2018) showed that a combination of DSCC and SCC could detect mastitis with greater accuracy and could more effectively monitor the various stages of infection. Halasa and Kirkeby (2020) also showed that the combination of DSCC and SCC with threshold values of 62% and 200,000 cells/mL, respectively, could lead to a reduction of antimicrobial use. In addition, recent studies revealed that DSCC may be able to detect changes in milk composition resulting from perturbation of mammary gland homeostasis due to inflammation or infection (Pegolo et al., 2021, 2022).

The possibility of differentiating between individual cell populations and measuring the percentages of different LYM subsets and of PMN and macrophages (**MAC**) may give a more accurate picture of the mammary gland response to mastitis infection. In this context, flow cytometry has been used to characterize the relative proportion of PMN, MAC, and LYM and is proposed as a valid tool to identify inflammatory processes in animals with low SCC (Rivas et al., 2001).

From the breeder's perspective, it is worth noting that different pathogens differently affect milk quality and cheese-making aptitude. In particular, *Prototheca* spp. infection seems to severely impair milk quality and technological characteristics, indicating a need to better understand the fundamental mechanisms underlying the mammary gland response to this pathogen (Pegolo et al., 2022). The aim of this study, therefore, was to investigate the associations between natural subclinical IMI from *Streptococcus agalactiae* and *Prototheca* spp. and milk differential cell counts assessed by cytofluorimetric analysis in Holstein cows.

Experimental procedures were approved by the Ethical Committee for Animal Welfare (OPBA – Organismo Preposto al Benessere degli Animali) of the Università Cattolica del Sacro Cuore and by the Italian Ministry of Health (protocol number 510/2019-PR of 19/07/2019). The study was conducted on Holstein cows belonging to one herd located in the Veneto region of northeastern Italy. The herd comprised more than 200 lactating cows and was selected on the basis of a survey previously carried out by the Experimental Zooprophylactic Institute of Venezie (**IZSVe**) to determine the herd prevalence of *Strep. agalactiae* and *Prototheca* spp. in the Veneto region. The cows were housed in freestalls and fed total mixed rations based mainly on corn silage, sorghum silage, and concentrates. Drinking water was available in automatic water bowls and milking was carried out twice a day. Animal health was managed by farmers and local veterinarians, who observed animals and performed exams when needed. Further details are reported in Pegolo et al. (2022). An initial bacteriological screening was performed on milk samples (50 mL, pools of 4 quarters) collected in accordance with National Mastitis Council guidelines (NMC, 2017) from 188 cows selected on the basis of the survey results. Three groups of animals were identified: negative at bacteriological examination and without a history of mastitis (n = 34); positive for *Strep. agalactiae* at bacteriological examination (n = 23); and positive for *Prototheca* spp. at bacteriological examination (n = 21). Information regarding the study cows (parity, DIM, milk yield, pregnancy status, health history) was extracted from herd management software (Dairy Comp Sata, Alta Italia Srl). A second assessment was performed 2 wk later to confirm the presence of the pathogen. Animals with clinical signs of mastitis and animals being treated with antibiotics were excluded from the trial. To have a sufficient number of animals for statistical inference and minimize the effect of some of the individual sources of variation, we applied a further filtering criterion and selected only animals >120 DIM and ≥2 parities, which then led us to create an experimental design with 47 cows (n = 20, negative; n = 13, positive for *Prototheca* spp.; n = 14, positive for *Strep. agalactiae*). Immediately after aseptic collection of milk samples for bacteriological analysis, approximately 100 mL of composite milk was manually collected, divided into 2 subsamples, and kept at 4°C. Bronopol was added to one subsample (50 mL), which was then transferred to the Milk Quality Laboratory of the Breeders Association of the Veneto Region (ARAV, Padua, Italy) for analysis of milk composition, SCC, and DSCC; the other aliquot was taken to the flow cytometry laboratory of the Department of Comparative Biomedicine, Food and Hygiene (BCA) of the University of Padua (Italy). Samples were processed within 24 h of collection for subsequent milk composition and cytofluorimetric analyses.

Microbiological analyses were carried out at the IZSVe. The milk samples were frozen after delivery to the laboratory and analyzed within 3 d. Aliquots of 10 µL of milk from composite samples were inoculated on each of the following selective media: Baird-Parker agar with rabbit plasma fibrinogen (BP-RPF; Biokar Diagnostics), tallium kristalviolette tossin agar (TKT; IZSVe internal production), and *Prothoteca* isolation medium (PIM; IZSVe internal production). Details of the microbiological analyses are reported in Pegolo et al. (2022).

Analyses of milk quality and composition, including protein, casein, fat, and lactose percentages and urea (mg/100 g), were carried out using an FT6000 MilkoScan infrared analyzer (Foss A/S). Somatic cell counts (cells/mL) and DSCC were determined with a Fossomatic 7 DC analyzer (Foss A/S). Then, SCC was log-transformed to SCS (Ali and Shook, 1980) to obtain a normal distribution. Two quantitative variables—PMN-LYM count and MAC count—were derived from the DSCC, taking into consideration total SCC, and log-transformed to achieve normality, as reported in Pegolo et al. (2021).

For the flow cytometric analyses, 50 mL of milk pooled from the 4 quarters of each selected cow was diluted 1:2 with sterile PBS, centrifuged at 365 × *g* for 15 min at 4°C, and the supernatant discarded; 50 mL of PBS was then added, the samples were centrifuged again, and the supernatant discarded. This procedure was performed twice to completely remove the fat from the samples. After these 3 washing steps, the cells were resuspended in 500 µL of RPMI (ThermoFisher Scientific) + sodium azide + fetal bovine serum (Merck KGaA). For each milk sample, 4 tubes were prepared containing (1) cells alone (without antibody, used as negative control); (2) anti cd4 antibody (**Ab**) conjugated with phycoerythrin (**PE**) and anti cd8 Ab conjugated with Alexa Fluor 647 (**AF647**); (3) anti cd11 Ab conjugated with fluorescein isothiocyanate (**FITC**) and anti cd14 Ab conjugated with PE; and (4) anti cd45 Ab conjugated with FITC, anti cd21 conjugated with PE, and anti cd18 Ab conjugated with AF647. A mixture of 50 µL of cells and 50 µL of diluted antibodies was placed in a flow cytometric tube and incubated in the dark for 30 min at 4°C. After incubation, 500 µL of PBS was added to each tube, which were then centrifuged at 208 × *g* for 10 min at 4°C, and the supernatant discarded. A further 900 µL of PBS was then added to each tube for the acquisition. Flow cytometric analyses were performed using a CyFlow Space flow cytometer (Partec-Sysmex, Sysmex Europe GmbH) fitted with a blue laser (488 nm), a red laser (635 nm), and a UV laser. Voltage settings were 151 for forward scatter (FSC), 347.5 for side scatter (SSC), 452 for FL1/FITC-conjugated Abs, 442.5 for FL2/PE-conjugated Abs, and 556 for FL5/Alexa Fluor647-conjugated antibodies. All detectors except FSC and SSC were set to logarithmic amplification. Flow cytometry acquisition was performed at a low flow rate and acquisition automatically stopped when the number of events in the R1 gate reached 20,000. The data were analyzed with the FlowMax software version 2.82 (Sysmex-Partec, Sysmex Europe GmbH). The morphology and complexity of the cells were evaluated in an FSC versus SSC dot plot; total white blood cells were identified as CD45+ CD18+ events; PMN as CD11b+ CD14− negative events; MAC as CD11b+ CD14+ events; T-helper lymphocytes (**THL**) as CD4+ CD8− events; T cytotoxic lymphocytes as CD8+ and CD4− events; and B lymphocytes as CD45+ CD21+ CD18+ events.

Before statistical analyses, animals with co-infection or changes in bacteriological status between the initial screening and sample collection were excluded. The SCC traits and individual leukocyte populations were analyzed by one-way ANOVA using the GLM procedure in SAS (SAS Institute Inc.). The explanatory variable was bacteriological status defined on 3 levels: (1) negative samples; (2) samples affected by *Strep. agalactiae*; (3) samples affected by *Prototheca* spp. Orthogonal contrasts (*P* < 0.05) were used to compare, first, negative versus positive samples at bacteriological examination (*Strep. agalactiae* and *Prototheca* spp.) to assess the effect of inflammation. A second level of contrast within positive samples compared the specific effects of infection caused by *Strep. agalactiae* versus *Prototheca* spp.

Mean milk production (± standard deviation) was 29.25 (±9.99) kg/d. Milk composition was 2.41 ± 1.74% fat, 3.49 ± 2.63% protein, 2.75 ± 0.28% casein, and 4.58 ± 0.45% lactose. The results of the ANOVA of the effects of bacteriological status on the investigated traits are reported in Table 1. Subclinical IMI (negative vs. positive on milk culture) strongly affected SCS (*P* < 0.001); DSCC was moderately affected when expressed as a percentage (*P* = 0.02) and strongly affected when expressed as a count (PMN-LYM and MAC counts, *P* < 0.001). Specifically, samples positive for *Strep. agalactiae* and *Prototheca* spp. had higher SCS (+61% and +49%, respectively) and DSCC values (+4% and +19%, respectively) than culture-negative samples (Figure 1). The effects of the 2 pathogens were more evident when DSCC was expressed as a log PMN-LYM count (+59% and +71%, respectively) or as a log MAC count (+63% and +72%, respectively) due to the inclusion of SCC in the calculation (Figure 1). The individual leukocyte populations (Figure 2) confirmed that mastitis infection increased the PMN proportion in culture-positive milk samples compared with culture-negative milk samples, particularly in the case of *Strep. agalactiae* infection (+51%). The 2 pathogens behaved in opposite ways with respect to MAC%: *Strep. agalactiae* increased MAC% by 41%, whereas *Prototheca* decreased it by 25%. *Prototheca* infection strongly increased the proportions of total T lymphocytes (**TL**; +87%) and THL (+83%) in the milk. Accordingly, the (PMN+MAC):TL ratio increased with *Strep. agalactiae* infection (+95%) and decreased with *Prototheca* infection (−43%) compared with culture-negative samples. The increase in the proportion of PMN+TL determined by cytofluorimeter in the *Strep. agalactiae* (+45%) and *Prototheca* (+62%) positive samples compared with the negative samples was higher than the corresponding increase in the DSCC proportion. The correlation between DSCC and PMN+TL was moderate to high (r = 0.55; data not shown), which suggests that the accuracy of the DSCC parameter as an udder health indicator depends on the type of causative agent.

**Table 1:** Analysis of variance results for the effect of bacteriological status^1^ for the investigated traits

Item^2^	Bacteriological status		Contrast *F*-value	
	*F*-value	*P*-value	Negative vs. positive	*Strep. agalactiae* vs. *Prototheca*
SCC traits				
SCS	22.63	<0.001	38.77***	4.11
DSCC, %	4.39	0.020	4.29*	3.77
log PMN-LYM count	21.18	<0.001	35.54***	4.48*
log MAC count	22.54	<0.001	40.30***	2.75
Leukocyte populations^3^				
Total WBC, %	16.67	<0.001	32.81***	0.07
PMN, %	10.23	0.001	9.86**	8.92**
MAC, %	11.98	0.001	2.45	20.20***
TL, %	4.14	0.024	2.36	6.57*
THL %	4.23	0.022	1.48	7.48**
CTL %	3.07	0.059	—	—
BL %	1.23	0.303	—	—
PMN+TL, %	10.24	0.001	17.53***	1.88
(PMN+MAC):TL, %	5.30	0.020	0.33	9.89**

1 Bacteriological status was classified into 3 classes: negative, positive for *Streptococcus agalactiae* and positive for *Prototheca.*

2 SCS = somatic cell score expressed as log_2_(SCC/100,000) + 3; DSCC = differential SCC; logPMN-LYM count = polymorphonuclear neutrophils-lymphocytes count expressed as log_2_[(PMN-LYM count)/100,000] + 3; logMAC count = macrophage count expressed as log_2_(MAC count/100,000) + 3; total WBC = total white blood cells, the sum of all individual leukocyte population percentages; BL = B lymphocytes; THL = T-helper lymphocytes; CTL = cytotoxic T lymphocytes; TL = total T lymphocytes.

3 Leukocyte populations are expressed as percentage of total events acquired by the cytofluorimeter.

* *P* < 0.05

** *P* < 0.01

*** *P* < 0.001.

**Figure 1:**
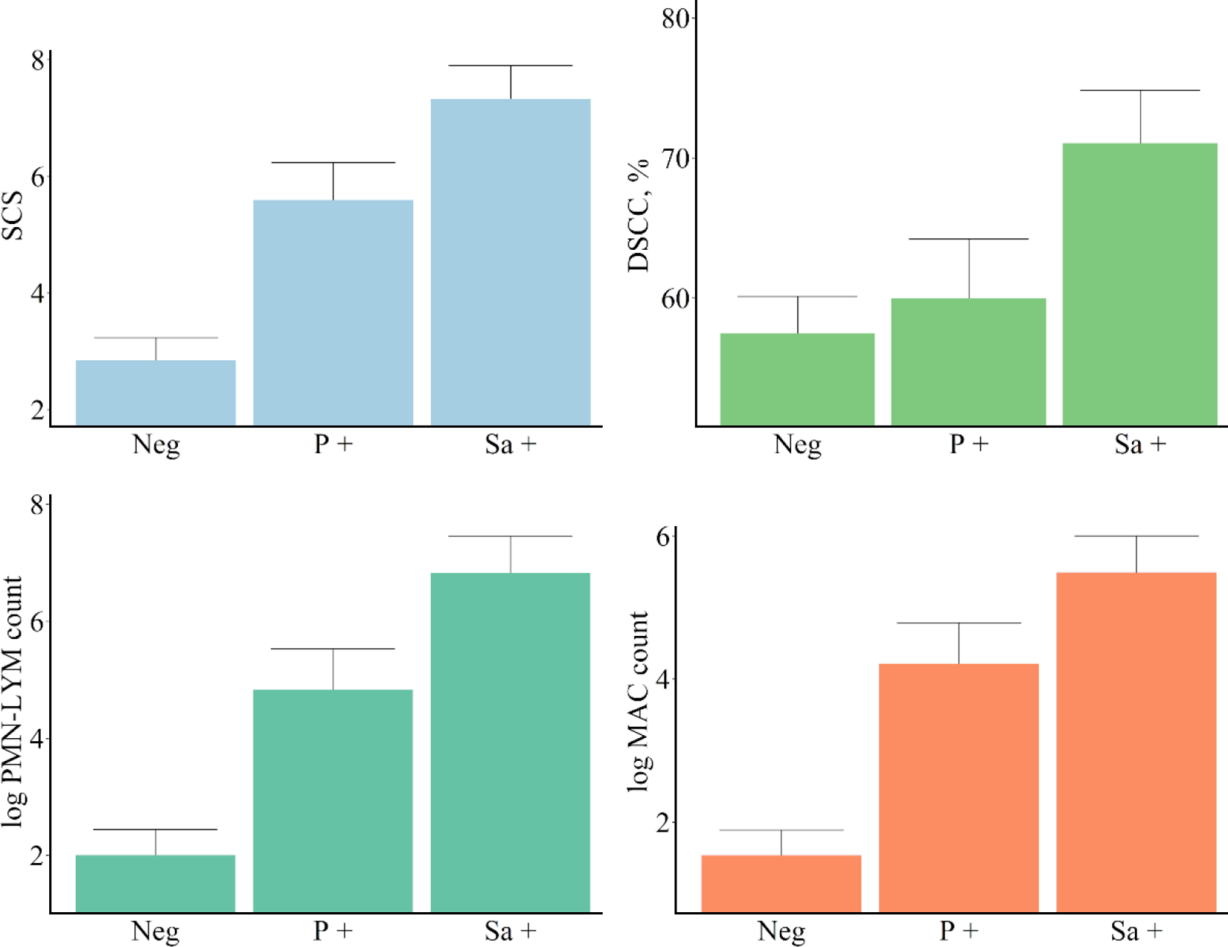
Least squares means (± SE) of SCC traits according to the bacteriological status. DSCC = differential SCC; PMN-LYM = polymorphonuclear neutrophils-lymphocytes; MAC = macrophages; logPMN-LYM count was expressed as log_2_[(PMN-LYM count)/100,000] + 3; macrophage (logMAC) count was expressed as log_2_(MAC count/100,000) + 3. Neg = samples negative at the bacteriological examination; Sa + = samples positive for *Streptococcus agalactiae* at the bacteriological examination; P + = samples positive for *Prototheca* at the bacteriological examination.

**Figure 2:**
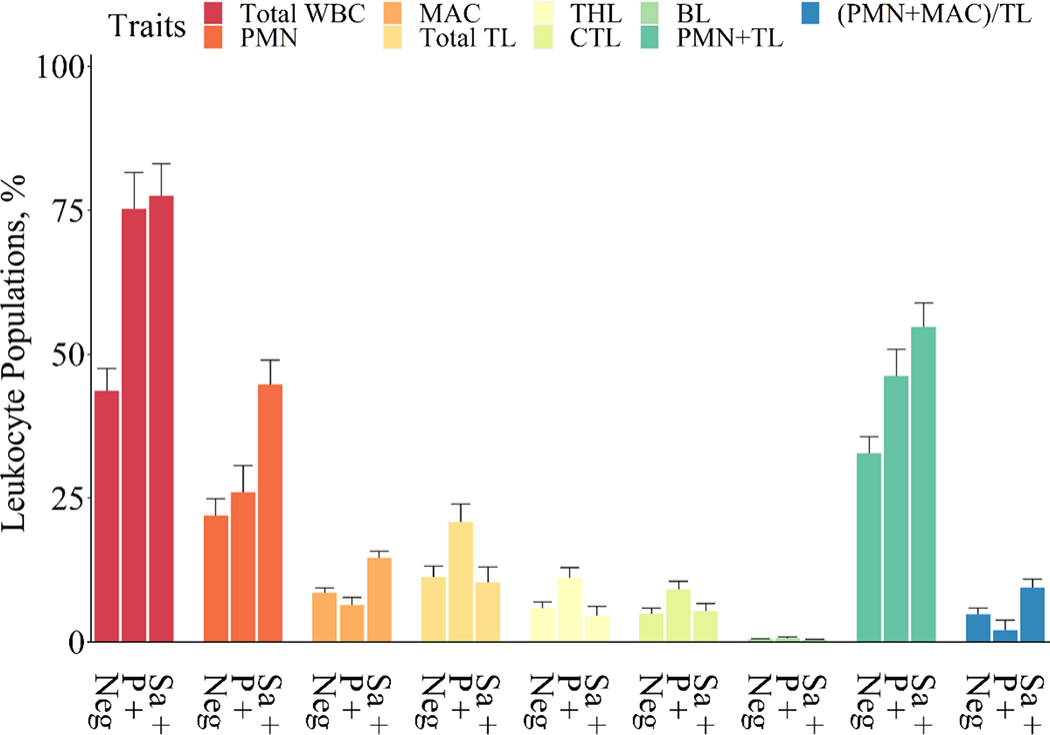
Least squares means (± SE) of leukocytes population according to the bacteriological status. Neg = samples negative at the bacteriological examination; Sa + = samples positive for *Strep. agalactiae* at the bacteriological examination; P + = samples positive for *Prototheca* at the bacteriological examination. WBC = white blood cells; BL = B lymphocytes; THL = T-helper lymphocytes; CTL = cytotoxic T lymphocytes; TL = total T lymphocytes; MAC = macrophages.

Our study showed that although *Strep. agalactiae* and *Prototheca* spp. infections are both responsible for a significant increase in SCC, they give rise to different profiles of leukocyte populations in milk somatic cells. As reported by Malinowski et al. (2006), a relationship exists between SCC and mastitis etiological agents. We detected higher SCC values in *Strep. agalactiae* infection than in *Prototheca* infection, in line with Zecconi et al. (2020). This might be because, unlike that of other mastitis pathogens, the initial immune reaction in the udder to *Prototheca* infection is mild and SCC usually remains unchanged during the initial stages of the disease. Most cases are subclinical, and infections become chronic when cows do not respond to routine therapy (Bueno et al., 2006; Wawron et al., 2013). We found that the dominant leukocyte populations in the mammary gland following *Strep. agalactiae* infection were PMN and MAC. These cell types represent the first line of response in the mammary gland to invading pathogens. In the healthy mammary gland, MAC predominate and act as sentinels to invading mastitis-causing pathogens. Once invaders are detected, MAC, and possibly mammary epithelial cells, release chemoattractants that direct a rapid influx of PMN to the area from the circulation. The release of oxidants by PMN is responsible not only for destroying bacteria but also for tissue damage, the latter limited by induction of apoptosis in PMN and engulfment by MAC migrating into the mammary gland through the pores of the capillaries (Paape et al., 2002). Higher neutrophil percentages may indicate more severe mastitis and a stronger innate immune response in the case of *Strep. agalactiae* infection compared with *Prototheca* infection.

Conversely, we found a higher proportion of lymphocytes, both TL and THL, in milk samples from animals infected with *Prototheca*. Many microorganisms have developed strategies to evade the host's innate immune response (Florea and Nightingale, 2004; Permpanich et al., 2006). Pathogenic *Prototheca* algae can form a biofilm to resist various environmental changes, which may be one of the factors in its pathogenicity. Cunha et al. (2006) found that neutrophils from bovine milk did not lead to the engulfment or death of *Prototheca zopfii*, despite an increased oxidative burst. Moreover, *P. zopfii* is able to modulate the host immune response: infected mammary gland epithelial cells increased expression of nuclear factor-κB p65 protein in their nuclei (Deng et al., 2016). The activation of this protein induces CD4 T-cell recruitment and differentiation in T-helper 1 and T-helper 17 cells, which mediate inflammatory responses against infections (Liu et al., 2017). In fact, the histological lesions caused by *P. zopfii* were characterized by granulomatous features with interstitial infiltrates of MAC, epithelioid and giant cells, plasma cells, and a thick infiltrate of LYM (Milanov et al., 2006). All of these features suggest the prevalence of an adaptive immune response and chronic inflammation in *Prototheca* infection, as opposed to the innate immune response to *Strep. agalactiae.* In line with the different features of infection, the (PMN+MAC):TL ratio was higher in samples positive for *Strep. agalactiae* than in those positive for *Prototheca*.

Our results have shown that *Strep. agalactiae* and *Prototheca* spp. trigger different immune responses in the bovine mammary gland. Identification of the specific *Prototheca* species is advisable, however. Additionally, different strains or genotypes of the pathogens could produce divergent results. Validation of these findings on a larger population and including multiple herds is therefore needed.

Finally, following the pattern of leukocyte populations over time could provide further information on stages of infection. Moreover, a quarter-level analysis could shed light on the association between the degree of inflammation and the leukocyte response. Improved knowledge of the immunological profile of the mammary gland against a particular pathogen will allow a better understanding of the nature, rate of development, and severity of mastitis caused by such a pathogen, and will lay the foundations for the development of novel and effective diagnostics and therapeutics.
